# Potential Molecular Markers Related to Lymph Node Metastasis and Stalk Resection Margins in Pedunculated T1 Colorectal Cancers Using Digital Spatial Profiling: A Pilot Study with a Small Case Series

**DOI:** 10.3390/ijms25021103

**Published:** 2024-01-16

**Authors:** Mi Jung Kwon, Ha Young Park, Hyun Lim, Il Tae Son, Min-Jeong Kim, Nan Young Kim, Min Jeong Kim, Eun Sook Nam, Seong Jin Cho, Woo Jin Bang, Ho Suk Kang

**Affiliations:** 1 Department of Pathology, Hallym University Sacred Heart Hospital, Hallym University College of Medicine, Anyang 14068, Republic of Korea; mulank@hanmail.net; 2 Department of Pathology, Busan Paik Hospital, Inje University College of Medicine, Busan 47392, Republic of Korea; 3 Department of Internal Medicine, Hallym University Sacred Heart Hospital, Hallym University College of Medicine, Anyang 14068, Republic of Korea; 4 Department of Surgery, Hallym University Sacred Heart Hospital, Hallym University College of Medicine, Anyang 14068, Republic of Korea; 5 Department of Radiology, Hallym University Sacred Heart Hospital, Hallym University College of Medicine, Anyang 14068, Republic of Korea; 6 Hallym Institute of Translational Genomics and Bioinformatics, Hallym University Medical Center, Anyang 14068, Republic of Korea; 7 Department of Surgery, Kangdong Sacred Heart Hospital, Gangdong-gu, Seoul 05355, Republic of Korea; 8 Department of Pathology, Kangdong Sacred Heart Hospital, Gangdong-gu, Seoul 05355, Republic of Korea; 9 Department of Urology, Hallym University Sacred Heart Hospital, Hallym University College of Medicine, Anyang 14068, Republic of Korea

**Keywords:** early colorectal cancer, submucosal invasion, T1, pedunculated, Haggitt level, lymph node metastasis

## Abstract

There is a debate regarding the prediction of lymph node metastasis (LNM) in pedunculated T1 colorectal cancer (CRC). In this study with four cases of pedunculated T1 CRCs, we aimed to investigate gene expression variations based on the distance from the Haggitt line (HL) and identify potential molecular risk factors for LNM. By leveraging the Cancer Transcriptome Atlas and digital spatial profiling technology, we meticulously analyzed discrete regions, including the head, HL, proximal stalk region (300–1000 μm from HL), and distal stalk region (1500–2000 μm from HL) to identify spatially sequential molecular changes. Our findings showed significant overall gene expression variations among the head, proximal stalk, and distal stalk regions of pedunculated T1 CRCs compared to the control adenoma. Compared to LNM-negative T1 CRCs, LNM-positive T1 CRC showed that the expression of genes involved in immune-related pathways such as *B2M*, *HLA*-*B*, and *HLA*-*E* were significantly downregulated in the distal stalk region compared to the proximal stalk region. In summary, our results may tentatively suggest considering endoscopic resection of the stalk with a minimum 2000 μm margin from the HL, taking into account the gene expression alterations related to immune-related pathways. However, we acknowledge the limitations of this pilot study, notably the small case series, which may restrict the depth of interpretation. Further validation is imperative to substantiate these findings.

## 1. Introduction

T1 colorectal cancers (CRC), which invade the submucosa, are categorized as early stage CRC [1], constituting around 17% of all CRC cases [2]. These T1 CRCs can be categorized into two primary morphological types: pedunculated and non-pedunculated, depending on the presence or absence of a stalk [3]. Among these, pedunculated T1 CRCs without lymph node metastasis (LNM) are particularly well-suited for endoscopic resection due to their higher likelihood of successful complete treatment and generally better prognosis compared to their non-pedunculated counterparts [4]. This is facilitated by the ease of performing en-bloc snare polypectomy for pedunculated T1 CRCs [5]. However, approximately 3–7% of patients with pedunculated T1 CRCs experience LNM, necessitating surgical resection with lymph node dissection following endoscopic treatment [6,7]. One notable challenge in diagnosing pedunculated T1 CRCs lies in accurately assessing submucosal invasion within these lesions during histopathological examination [8]. Measuring the depth of submucosal invasion is relatively straightforward in non-pedunculated T1 CRC, where it is typically assessed by measuring the distance from the lamina propria; however, evaluating submucosal invasion in pedunculated T1 CRC can present a relatively complicated challenge due to the intricate invagination of the submucosal layer throughout various regions of the polyp, including the head, neck, and stalk [9].

To address this challenge, the Haggitt classification system, introduced in 1985, categorizes pedunculated T1 CRCs into “head (level 1)”, “neck (level 2)”, “stalk (level 3)”, and “beyond stalk (level 4)” to classify the depth of submucosal invasion [10]. In pedunculated T1 CRCs, the Haggitt line, representing the neck (level 2), serves as a critical reference point, with invasion from the Haggitt line to the stalk (level 3) defining the depth of submucosal invasion [10]. Importantly, research findings have revealed that even pedunculated T1 CRCs classified within levels 1 (13.3%) to level 3 (10.9%) according to the Haggitt classification system can still manifest LNM [11] or distant metastasis to the liver within 3 years in completely resected level 1 pedunculated T1 CRC [12]. These findings challenge the conventional assumption that lower Haggitt levels inherently indicate a low or negligible risk of LNM [10]. Moreover, these observations contradict prior notions that pedunculated early invasive CRCs pathologically diagnosed as head invasion can be effectively managed solely through endoscopic treatment [6]. Distinguishing the Haggitt line can also be a complex task, further complicated by variations in the length of the stalk among patients. Data regarding pedunculated T1 CRCs remain relatively scarce in comparison to the available data for non-pedunculated T1 CRCs [13]. Thus, compared to the straightforward risk factor of deep submucosal invasion (≥1000 µm) for LNM in non-pedunculated T1 CRCs in endoscopic specimens [14], further evaluation is required to understand the impact of submucosal invasion depth from the Haggitt line on the risk of LNM in pedunculated T1 CRCs.

As histological examination alone may not always accurately define the tumor’s extent, especially in precancerous lesions around the visibly identifiable tumor mass [15], we turn to new technologies such as the digital spatial profiling assay. This innovative multiplexed immunohistochemistry assay allows for the simultaneous detection and spatial analysis of multiple proteins or RNAs within a single tissue section or between different tissue samples, offering valuable spatial information ranging from the subcellular to the cell population level [16].

In this study, we utilize digital spatial profiling technology to investigate the sequential molecular changes in the head, neck, and stalk of pedunculated T1 CRCs. This approach enables us to identify gene expression differences according to the Haggitt line and to explore molecular risk markers associated with LNM, providing valuable insights into the spatial characteristics of these lesions.

## 2. Results

Table 1 provides a summary of the four cases included in this study. Among them, there were three male and one female patient, with an average age of 72.5 years (range: 68–76 years). All four cases presented well to moderately differentiated CRC tumors measuring smaller than 20 mm in size. Haggitt line invasion was observed in all cases, with stalk invasion from the Haggitt line being less than 1000 μm in two cases and more than 1000 μm in the remaining two cases. Lymphovascular invasion was positive in all cases, and LNM was detected in one case. None of these patients experienced recurrence during the follow-up period, which averaged 2803 days (range: 1169–3997 days).

### 2.1. The Overall Gene Expression Pattern Related Distance from Haggitt Line

For a more in-depth analysis, within the pedunculated T1 CRC cases (including cases 1, 2, 3, and 4), we categorized them into the following groups: the LNM-positive group (represented by case 4), the LNM-negative group (comprising cases 1, 2, and 3), the proximal stalk invasion group (cases 2 and 3), and the distal stalk invasion group (cases 1 and 4).

**Figure 1:**
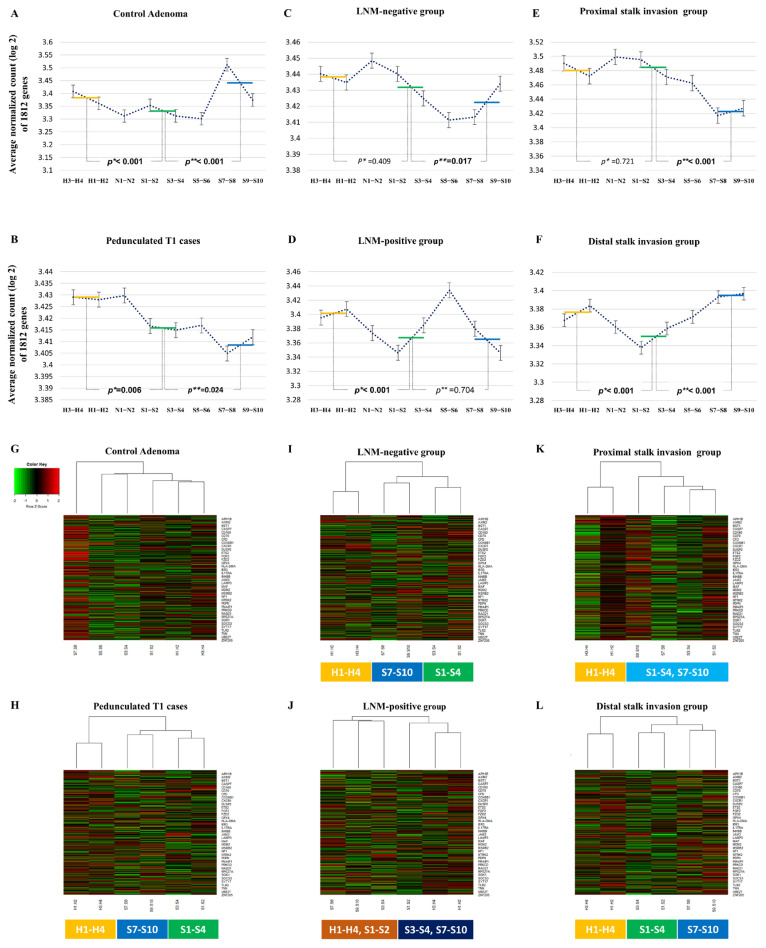
Comprehensive analysis of the average normalized count of 1812 genes (the sum of log2 normalized counts of 1812 Cancer Transcriptome Atlas genes, divided by 1812) across different ROIs, categorized into three regions based on their proximity to the Haggitt line: the head region (ROIs H1–H4, yellow line), proximal stalk region (ROIs S1–S4, green line), and distal stalk region (ROIs S7–S10, blue line). (**A**) The control adenoma exhibited higher gene expression in the head region compared to the proximal stalk region, with a subsequent increase in gene expression observed in the distal stalk region compared to the proximal stalk region. (**B**) Within the pedunculated T1 CRC group (cases 1, 2, 3, and 4), a decrease in gene expression from the head to the proximal stalk region was noted, followed by an increase in gene expression from the proximal to the distal stalk region. (**C**) In the LNM-negative group (cases 1, 2, and 3), gene expression was higher in the proximal stalk region compared to the distal stalk region. (**D**) Conversely, in the LNM-positive group (case 4), gene expression was lower in the proximal stalk region than in the head region. (**E**) Within the proximal stalk invasion group (cases 2 and 3), gene expression was higher in the proximal stalk region than in the distal stalk region. (**F**) In the distal stalk invasion group (cases 1 and 4), gene expression exhibited a decrease from the head to the proximal stalk region and was lower than in the distal stalk region. (**G**–**L**) Hierarchical clustering analysis of 1812 genes according to three regions (head region, proximal stalk region, and distal stalk region) are shown. Each row corresponds to a specific gene (among the 1812 genes, only some gene names are indicated), while each column represents an ROI. Pedunculated T1 cases (**H**), LNM-negative group (**I**), and distal stalk invasion group (**L**) have three clusters according to three regions identified, whereas the proximal stalk invasion group (**K**) was clustered into the head region and stalk region (proximal and distal). Within the LNM-positive group (**J**), two clusters were divided around the center of the proximal stalk region. p* represents the value obtained by comparing the head region (ROIs H1–H4) and proximal stalk region (ROIs S1–S4). p** represents the value obtained by comparing the distal stalk region (ROIs S7–S10) and proximal stalk region.

To identify candidate regions where 1812 Cancer Transcriptome Atlas genes differences significantly between ROI clusters of pedunculated T1 CRC cases, we performed a comprehensive analysis of the average normalized count of 1812 genes across different ROIs, categorizing them based on their proximity to the Haggitt line (Figure 1A–F). In the control adenoma, gene expression in the ROIs S1–S4 was significantly lower than in the ROIs H1–H4, and there was a significant increase in gene expression in the ROIs S7–S10 compared to the ROIs S1–S4. Within the pedunculated T1 CRC cases, there was a significant reduction in gene expression in the ROIs S1–S4 compared to the ROIs H1–H4 and an increase in gene expression from the ROIs S1–S4 to the distal stalk region. In the LNM-negative group, gene expression in the ROIs S1–S4 was notably higher than in the ROIs S7–S10. Conversely, in the LNM-positive group, gene expression in the ROIs S1–S4 was significantly lower than in the ROIs H1–H4. Within the proximal stalk invasion group, gene expression in the ROIs S1–S4 was markedly higher than in the ROIs S7–S10. In the distal stalk invasion group, there was a significant reduction in gene expression in the ROIs S1–S4 compared to the ROIs H1–H4 and a reduction compared to the distal stalk region. Therefore, we considered the possibility that there were changes in overall gene expression before and after the ROIs S1–S4, and categorized the ROIs into three ROI regions: the head region (the ROIs H1–H4), proximal stalk region (the ROIs S1–S4), and distal stalk region (the ROIs S7–S10). Furthermore, hierarchical clustering analysis (Figure 1G–L) was performed to evaluate the correlation between 1812 gene expression in the head region, proximal stalk region, and distal stalk region. Unlike control adenoma, in pedunculated T1 cases, the LNM-negative group, and distal stalk invasion group, gene expressions were clearly clustered by each of the three regions. The proximal stalk invasion group was clustered into the head region and stalk region (proximal and distal), and the LNM-positive group was classified into two clusters based on the center of the proximal stalk region. Additional research was carried out to determine the differentially expressed genes that create this cluster pattern by comparing cases.

### 2.2. Differentially Expressed Genes Associated with Lymph Node Metastasis

**Figure 2:**
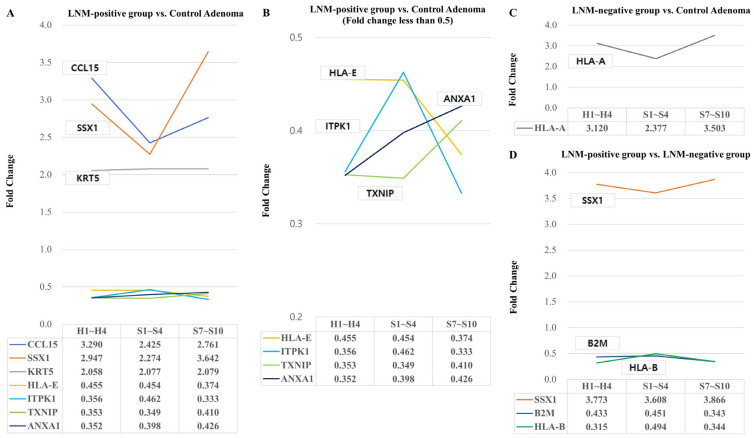
Differentially expressed genes associated with lymph node metastasis across the head region (ROIs H1–H4), proximal stalk region (ROIs S1–S4), and distal stalk region (ROIs S7–S10) were examined. A fold change > 2 or fold change ≤ 0.5, and *p*-values < 0.05 were considered significant differentially expressed genes.

In the LNM-positive group compared to the control adenoma, there were significant increases in gene expression for *CCL15*, *SSX1*, and *KRT5* genes (Figure 2A), alongside significant decreases in *HLA*-*E*, *ITPK1*, *ANXA1*, and *TXNIP* genes (Figure 2B) across the head, proximal stalk, and distal stalk regions.

In the LNM-negative group compared to the control adenoma, there was a significant increase in the gene expression of the *HLA*-*A* gene (Figure 2C).

Additionally, in the LNM-positive group compared to the LNM-negative group, we observed a significant increase in *SSX1* gene expression and a decrease in *B2M* and *HLA*-*B* gene expression (Figure 2D).

### 2.3. Differentially Expressed Genes Associated with Proximal and Distal Stalk Invasion

**Figure 3:**
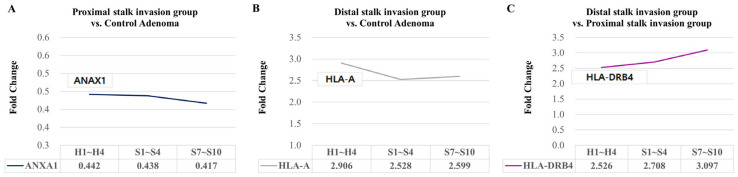
Differentially expressed genes associated with deep stalk invasion (≥1000 μm) across the head region (ROIs H1–H4), proximal stalk region (ROIs S1–S4), and distal stalk region (ROIs S7–S10) were examined. A fold change > 2 or fold change ≤ 0.5 and *p*-values < 0.05 were considered significant differentially expressed genes.

In the proximal stalk invasion group compared to the control adenoma, we observed a significant decrease in *ANAX1* gene expression across the head, proximal stalk, and distal stalk regions (Figure 3A).

In the distal stalk invasion group compared to the control adenoma, there was a significant increase in *HLA*-*A* gene expression (Figure 3B).

Additionally, when comparing the distal stalk invasion group to the proximal stalk invasion group, we found a significant increase in *HLA*-*DRB4* gene expression (Figure 3C).

### 2.4. KEGG Pathway Analysis of Differentially Expressed Genes

While genes like *CCL15*, *SSX1*, *KRT5*, and *HLA*-*A* showed significantly increased expression levels in relation to LNM in this study, the KEGG pathway analysis did not yield significant results for these genes. Similarly, despite genes *HLA*-*A* and *HLA*-*DRB4* displaying significantly elevated expression levels in relation to distal stalk invasion in this study, the KEGG pathway analysis did not yield significant results for these genes.

Notably, when we examined the genes with reduced expression in LNM, including *HLA*-*E*, *ITPK1*, *TXNIP*, *ANXA1*, *B2M*, and *HLA*-*B*, the KEGG pathway analysis revealed significant results in multiple pathways, particularly those related to antigen processing and presentation. The top 10 significant pathways associated with the expression of *HLA*-*E*, *B2M*, and *HLA*-*B* genes are displayed (Table 2).

### 2.5. Changes in HLA-E, B2M, and HLA-B According to Lymph Node Metastasis or Stalk Invasion

**Figure 4:**
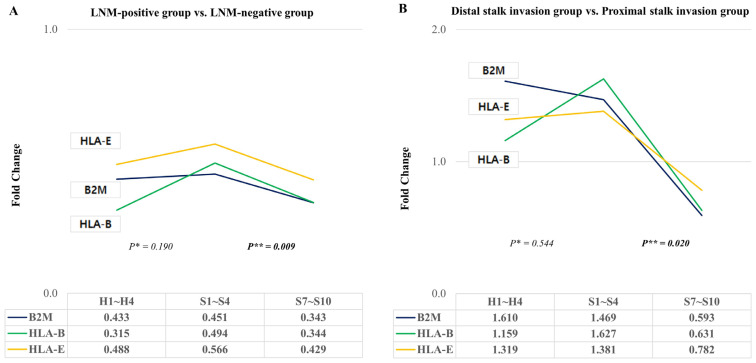
Changes in *HLA*-*E*, *B2M*, and *HLA*-*B* according to lymph node metastasis and submucosal invasion were analyzed. p* represents the value obtained by comparing the head region (ROIs H1–H4) and proximal stalk region (ROIs S1–S4). p** represents the value obtained by comparing the distal stalk region (ROIs S7–S10) and proximal stalk region.

When comparing the fold change in gene expression levels of *B2M*, *HLA*-*B*, and *HLA*-*E* genes between the LNM-positive and LNM-negative groups, we observed an increase in the proximal stalk region compared to the head region, although this difference was not statistically significant (*p* = 0.190). Notably, there was a significant reduction in the distal stalk region compared to the proximal stalk region in the LNM-positive group (*p* = 0.009) (Figure 4A).

In the comparison of the fold change in gene expression levels of *B2M*, *HLA*-*B*, and *HLA*-*E* genes between the distal stalk invasion and proximal stalk invasion groups, we observed an increase in the proximal stalk region compared to the head region, but this difference did not reach statistical significance (*p* = 0.544). However, we did find a significant reduction in the distal stalk region compared to the proximal stalk region in the distal stalk invasion group (*p* = 0.020) (Figure 4B).

## 3. Discussion

Our goal was to investigate gene expression profile variations among the tumor head, proximal stalk (300–750 μm from Haggitt line), and distal stalk regions (1500–2000 μm from Haggitt line) and assess these spatial molecular changes, particularly in relation to LNM and distal stalk invasion in pedunculated T1 CRCs. We found significant differences in gene expression levels among the head, proximal stalk, and distal stalk regions within both the control and pedunculated T1 CRC groups. These differences were consistent across various patient subgroups, including LNM-positive, LNM-negative, proximal stalk invasion, and distal stalk invasion groups. These findings highlight the importance of considering the specific anatomical regions within pedunculated T1 CRCs when assessing gene expression patterns, as this can offer insights into the spatial heterogeneity of these tumors.

We observed significant downregulation in the gene expression of *B2M*, *HLA*-*B*, and *HLA*-*E* genes in the distal stalk region compared to the proximal stalk region in the LNM-positive group. Pathway analysis indicated significant alterations in immune-related pathways, particularly those related to antigen processing and presentation, driven by changes in *HLA*-*E*, *B2M*, and *HLA*-*B* gene expression. The antigen presentation pathway plays a pivotal role in the body’s immune surveillance against cancer cells [17]. It enables the immune system to recognize and target abnormal or malignant cells effectively [17]. Our study underscores the potential significance of *HLA*-*I* (human leukocyte antigen class I) genes, including *HLA*-*E*, *B2M*, and *HLA*-*B* [18], in the context of LNM of pedunculated T1 CRCs. The downregulation of genes such as *HLA*-*E*, *B2M*, and *HLA*-*B*, which are integral components of the antigen presentation pathway, suggests a potential immune evasion mechanism in pedunculated T1 CRCs with LNM. When these genes are downregulated, it may impair the tumor cells’ ability to present antigens to immune cells, thus hindering the immune system’s capacity to detect and eliminate the cancer. In the context of cancer, *HLA*-*I* molecules play a critical role in presenting tumor-derived antigen peptides to cytotoxic T lymphocytes, thereby contributing significantly to cancer cell eradication [18]. However, in various cancer types, including CRC, these molecules are often lost, leading to immune evasion during cancer development. This loss of *HLA*-*I* is attributed to accumulating mutations and heterozygosity loss at the *HLA*-*I* and *B2M* loci [19], which poses a significant challenge for developing effective anti-tumor immunotherapies by rendering T cells without targets. Notably, a meta-analysis in gastrointestinal cancer has shown that *HLA*-*I* overexpression is linked to improved prognosis [20], whereas *HLA*-*I* loss in tumors is a key mechanism for evading T cell-mediated responses [21,22,23,24,25] associated with therapeutic failure and unfavorable prognosis indicating initial resistance and secondary immune escape after immunotherapy such as anti-PD-1/PD-L1 [19]. In this context, our analysis reveals a notable decrease in the expression of *HLA*-*I*-related genes within 1000 μm from the Haggitt line in cases with LNM and deep stalk invasion, suggesting that this reduction might be linked to immune escape mechanisms in the occurrence of LNM of pedunculated T1 CRCs. These findings may hold potential promise for guiding future research into targeted immunotherapies tailored for pedunculated T1 CRCs with LNM. Strategies aimed at restoring or enhancing antigen presentation in these cases may offer potential avenues to improve the immune response and, ultimately, patient outcomes.

The clinical significance of distal stalk invasion as an independent predictor for LNM in pedunculated T1 CRCs has been debated, with some studies emphasizing its importance [26,27,28,29] and others questioning its isolated significance [11,30]. A recent meta-analysis did not find distal stalk invasion to be a significant predictor for LNM (odds ratio, 1.73; 95% confidence intervals, 0.96–3.12) [30]. Notably, in the context of pedunculated T1 CRC, a study indicated no significant difference in the LNM rate between cases with “head invasion” (4/30, 13.3%) and “stalk invasion” (5/46, 10.9%) [11]. Given the challenges associated with achieving Haggitt level 4 resection during endoscopic procedures, it is common practice for endoscopists to opt for resection through the stalk rather than attempting to reach Haggitt level 4, as there is no established minimum endoscopic resection margin requirement from the Haggitt line [14]. In such clinical scenarios, complete resection of pedunculated malignant polyps with level 1–3 invasion is typically considered adequate, and additional surgery is generally unnecessary, provided there are no additional risk factors such as lymphovascular invasion or poorly differentiated histology [31]. However, our findings reveal significant gene expression changes occurring within the range of 1000–2000 μm from the Haggitt line in cases with both LNM and distal stalk invasion. This underscores the critical importance of considering the entire stalk when assessing pedunculated T1 CRCs. Accurate assessment of submucosal invasion depth within pedunculated T1 CRCs is critical for determining the appropriate treatment strategy, particularly in distinguishing cases that can be effectively managed through endoscopic resection from those requiring surgical intervention. As a result, our study may recommend resecting the stalk with a margin of at least 2000 μm from the Haggitt line, even when complete endoscopic resection has been achieved.

Recent research comparing normal mucosa, low-grade dysplasia, high-grade dysplasia, and cancer in eight endoscopic submucosal dissected T1 CRC lesions also utilized digital spatial profiling [32]. This study observed gradual changes in immune cell composition from normal tissue to areas of dysplasia and cancer, reinforcing our findings regarding immune response involvement in T1 CRC progression [32]. However, our study specifically focused on changes occurring beneath the lamina propria and submucosa of the pedunculated type. Furthermore, we conducted a detailed analysis of sequential changes within ROIs and compared the average values of two or more ROIs in one area per case to ensure statistical significance.

The limitation of the present study was the limited number of patients who were only four pedunculated T1 CRC with full confirmed specimens through complete endoscopic resection, subsequent surgery, and lymph node dissection and the limited study design as a pilot study. Although the patient number enrolled in the study appears small, the cases fully confirmed through endoscopic resection, surgery, and lymph node dissection were indeed minor, and the specimens examined in the study were well defined. Our results should be confirmed through a larger case cohort in future multicenter studies. Specifically, further research is needed to ascertain whether the alterations in *HLA*-*E*, *B2M*, and *HLA*-*B*, as indicated in our findings, are indeed linked to cancer immune evasion through the loss of MHC Class I antigen presentation. Furthermore, in light of a recent study demonstrating that CRC is associated with an immunosuppressive microenvironment characterized by alterations in macrophage and lymphocyte infiltration [17], it is imperative that our research findings undergo additional validation through immune context analysis. This additional investigation is essential for confirming the intricate and dynamic interplay between tumor cells and the immune system.

Nonetheless, our study may contribute to the understanding of the correlation of gene expression profile among the tumor head, proximal stalk (300–750 μm from Haggitt line), and distal stalk regions (1500–2000 μm from Haggitt line) and assess these spatial molecular changes, particularly in relation to LNM and distal stalk invasion in pedunculated T1 CRCs, a field that is not yet well elucidated. To overcome the limitations associated with bulk and single-cell approaches, we adopted a digital spatial profiling technique, which may be another strength of our study. This approach allowed us to profile discrete regions of pedunculated T1 CRCs, providing transcriptome information while preserving spatial data on tissue morphology and structure. We utilized digital spatial profiling with Cancer Transcriptome Atlas probes to analyze 1812 RNA targets in all regions of interest within a single tissue section. Notably, our study represents the first in situ molecular genetic analysis of pedunculated T1 CRCs, considering the Haggitt level and its association with LNM.

## 4. Materials and Methods

### 4.1. Patients and Histologic Evaluation

We reviewed 2718 cases of CRC surgery at Hallym University Sacred Heart Hospital in Anyang, South Korea, from October 2010 to March 2021. We focused on 10 cases of pedunculated T1 CRC patients who had both endoscopic mucosal resection and subsequent bowel resection with lymphadenectomy. We excluded cases that lacked endoscopic images or accessible pathological specimens before and after surgery, as well as those involving sessile serrated lesions. Additionally, specimens that did not contain sufficient submucosal layer to produce sufficient ROIs or were damaged during the experimental slide manufacturing process were excluded. Ultimately, we included four eligible cases in our study and added one pedunculated tubular adenoma with low-grade dysplasia as a control group. The endoscopic mucosal resection specimens were processed following institutional guidelines, involving fixation in formalin, gross sectioning, embedding in paraffin blocks, slicing in 4-μm thickness, and H&E staining. A gastrointestinal pathologist (MJK) microscopically confirmed various parameters, including histologic type, resection margins, invasion depth, lymphovascular invasion, and the Haggitt line. Patients provided informed consent before endoscopic mucosal resection, and the study was approved by the institutional review board of Hallym University Sacred Heart Hospital (approval number 2021-11-006-003), conducted following the Declaration of Helsinki and relevant guidelines.

### 4.2. Digital Spatial Profiling and nCounter Data Analysis

The GeoMx^®^ Cancer Transcriptome Atlas probe reagent (GMX-RNA-NGS-CTA-4, Nanostring, Seattle, WA, USA) was introduced to slide samples following Nanostring’s standard protocol (MAN-10115-05 for software v2.5, Nanostring, Seattle, WA, USA). This Cancer Transcriptome Atlas probe is designed to simultaneously profile over 1812 RNA targets with spatial resolution. It utilizes in situ hybridization probes that are conjugated to unique DNA indexing-oligonucleotides through a UV-photocleavable linker. Additionally, fluorescently labeled markers, including pan-CK (GMX-RNA-MORPH-HST-12, Nanostring, Seattle, WA, USA) for detecting tumor cells, Ki-67 (sc-23900, Santacruz, Dallas, TX, USA) for confirming cell proliferation, anti-desmin antibody (ab203419, Abcam, Cambridge, UK) for staining the lamina propria, and SYTO-13 (GMX-RNA-MORPH-HST-12, Nanostring, Seattle, WA, USA) for counterstaining nuclear DNA, were applied to 4 μm thick FFPE tissue section slides. After incubation, these slides were loaded onto an inverted microscope within NanoString’s GeoMx Digital spatial profiling instrument. These slides were scanned to capture fluorescent images, which were then used to identify regions of interest (ROI). The size of these ROIs typically ranged from 300 μm in diameter, with 16–18 ROIs selected per slide, depending on the researcher’s specific focus (MJK, HSK), primarily based on morphology.

**Figure 5:**
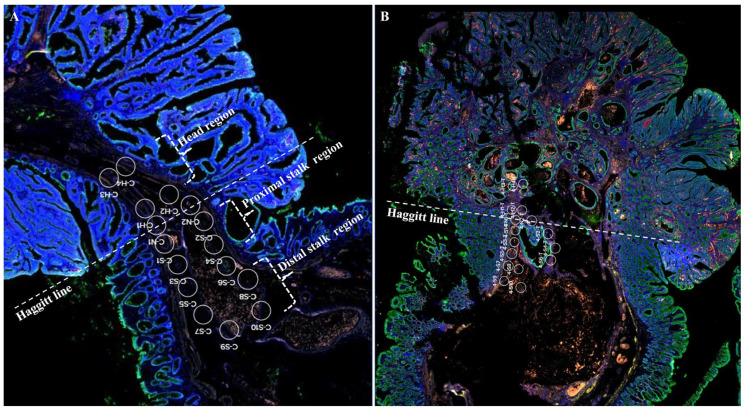
Regions of interest (ROIs) were selected for molecular profiling analysis from the slides of a pedunculated tubular adenoma with low-grade dysplasia as a control (**A**) and pedunculated T1 colorectal cancers (**B**). These slides were imaged using four antibodies (pan-cytokeratin in green, Ki-67 in red, Anti-Desmin antibody in yellow, and SYTO-83 in blue) to visualize tissue morphology. The ROIs encompass Haggitt line (neck line, N1–N2); 1000 μm and 300 μm from the Haggitt line in the direction of the tumor head (head region; H3–H4 and H1–H2); 300 μm (proximal stalk region; S1–S2), and 750 μm (proximal stalk region; S3–S4), 1000 μm (stalk region; S5–S6), 1500 μm (distal stalk region; S7–S8), and 2000 μm (distal stalk region; S9–S10) away from the Haggitt line in the direction of the tumor stalk.

To assess the submucosal microenvironment in the context of Haggitt’s lines, two ROIs (neck line; N1, N2) were selected visually in the submucosa near the lamina propria. Similarly, ROIs were selected along parallel imaginary lines at 300 μm intervals (head portion; H1, H2) and 1000 μm intervals (H3, H4) away from the Haggitt line, in the direction of the tumor’s head portion. Additionally, ROIs were designated at 300 μm intervals (stalk portion; S1, S2), 750 μm intervals (S3, S4), 1000 μm intervals (S5, S6), 1500 μm intervals (S7, S8), 2000 μm intervals (S9, S10), and 3000 μm intervals (S11, S12) away from the Haggitt line, in the direction of the tumor’s stalk portion (Figure 5).

Once the ROIs were selected, a programmable digital micromirror device precisely directed UV light to illuminate each ROI, cleaving oligos in a region-specific manner. The released indexing DNA oligonucleotides were then collected through microcapillary aspiration and deposited into individual wells of a microtiter plate. This process of localized UV exposure and UV cleaved-oligo collection was repeated for each ROI. The collected indexing oligos were subsequently hybridized to NanoString’s fluorescent barcodes, enabling the digital counting of binding events, with the capacity to count up to 1 million events per ROI, using the NanoString nCounter Analysis system.

For data normalization, the raw counts of indexing-oligos for ROIs were initially processed using NanoString’s third quartile (Q3) normalization procedure according to the manufacturer’s recommendations (MAN-10154-01-GeoMx-DSP-Data-Analysis, Nanostring, Seattle, USA) without selecting housekeeping genes [33]. Q3 normalization divides the raw counts in one ROI by the 3rd quartile value for that ROI, then subsequently multiplies that value by the geometric mean of the 3rd quartile values of all ROIs. Quality control measures were applied, including generating relative log expression plots based on the Q3 and median of ratios normalized counts, as part of the exploratory data analysis.

### 4.3. Statistical Analyses

The cases were categorized into distinct groups based on the presence of LNM and the depth of invasion from the Haggitt line. Specifically, cases with proximal stalk invasion, defined as invasion depth <1000 μm from the Haggitt line, were grouped in the “proximal stalk invasion group”. On the other hand, cases with distal stalk invasion, defined as invasion depth ≥1000 μm from the Haggitt line, were classified in the “distal stalk invasion group”. Additionally, cases with LNM were placed in the “LNM-positive group”, while cases without LNM were grouped in the “LNM-negative group”.

To identify significant differences in gene expression based on the distance from the Haggitt line within each of these groups, we categorized ROIs either individually or collectively and conducted comparisons. In these ROI regions, we evaluated the average normalized count of 1812 genes (the sum of log2 normalized counts of 1812 Cancer Transcriptome Atlas genes, divided by 1812), and differences in normalized counts were assessed using paired T-tests. Differentially expressed genes between groups were identified using a two-sample T-test, considering a fold change ≥ 2 and *p*-values < 0.05 as criteria for significant increase and a fold change ≤ 0.5 and *p*-values < 0.05 as criteria for significant decrease. Hierarchical clustering was performed using the R package, version 4.13, using Pearson’s correlation.

For statistical analysis, we utilized SPSS 21.0 software (IBM-SPSS Inc., Chicago, IL, USA) and ExDEGA (Ebiogen, Seoul, Republic of Korea). To gain insights into the biological pathways associated with these differentially expressed genes, we performed KEGG (Kyoto Encyclopedia of Genes and Genomes) pathway analysis using G:profiler (https://biit.cs.ut.ee/gprofiler/gost (accessed on 27 June 2023)), and we employed the false discovery rate adjustment method by Benjamini–Hochberg for the adjusted *p*-values [34].

## 5. Conclusions

This study may present preliminary evidence supporting the recommendation of stalk resection with a margin of at least 2000 μm from the Haggitt line in pedunculated T1 CRC cases. Utilizing digital spatial profiling technology to delve into the molecular characteristics of pedunculated T1 CRCs, with a focus on gene expression patterns related to the Haggitt line, LNM, and stalk invasion, our findings may highlight the spatial heterogeneity of these lesions for gene expression profile. Notably, immune-related genes, particularly *HLA*-*I* genes such as *HLA*-*E*, *HLA*-*B*, and *B2M*, appear to be potential players in cases with LNM. Since our study is a pilot study with a small case series focusing on four eligible pedunculated T1 CRC cases, further research is necessary to elucidate the functional significance of the identified differentially expressed genes and their potential roles as molecular markers for risk stratification and treatment decision making in pedunculated T1 CRCs. Moreover, exploring the implications of altered antigen processing and presentation pathways in these tumors may open up new avenues for future therapeutic interventions.

## Figures and Tables

**Table 1:** Demographic and clinical features of patients with pedunculated T1 colorectal cancers.

Patient	Sex	Age (y)	EMR Histology						cTNM	Op	LN Status	pTNM	Recurrence
			Location	Size (mm)	Grade	EMR Margin	Invasion Depth	LVI					(F/U Days)
1	F	71	Distal sigmoid	10	Well	Clear	1000 µm from HL	+	cT1N0M0	LAR	0/5	pT1N0M0	No (3997)
2	M	76	Distal sigmoid	13	Moderately	Clear	500 µm from HL	+	cT1N0M0	LAR	0/3	pT1N0M0	No (3935)
3 *	M	75	Transverse colon	17	Moderately	Clear	300 µm from HL	+	cT1N0M0	LH	0/17	pT1N0M0	No (2114)
4	M	68	Mid sigmoid	15	Well	Clear	1000 µm from HL	+	cT1N0M0	LAR	1/12	pT1N1aM0	No (1169)

M, male; F, female; EMR, endoscopic mucosal resection; Well, well differentiated; Moderately, moderately differentiated; LVI, lymphovascular invasion; HL, Haggitt line; cTNM, clinical TNM staging; Op, operation; LAR, lower anterior resection, LH, left hemicolectomy; LN, lymph node; pTNM, pathological TNM staging; F/U, follow up. * As Case 3 presented an additional non-pedunculated sigmoid colon cancer with submucosal invasion measuring 1 cm in size, a left hemicolectomy was conducted. This procedure allowed for the identification of a greater number of total lymph nodes.

**Table 2:** KEGG pathway analysis of genes (*HLA*-*E*, *ITPK1*, *TXNIP*, *ANXA1*, *B2M*, and *HLA*-*B*) associated with lymph node metastasis in pedunculated T1 colorectal cancers, displaying the top 10 signaling pathways.

Signaling Pathway	*p*-Value	Related Genes	KEGG ID
Antigen processing and presentation	0.000137	*HLA*-*E*, *B2M*, *HLA*-*B*	KEGG:04612
Human T-cell leukemia virus 1 infection	0.00068	*HLA*-*E*, *B2M*, *HLA*-*B*	KEGG:05166
Allograft rejection	0.00068	*HLA*-*E*, *HLA*-*B*	KEGG:05330
Human immunodeficiency virus 1 infection	0.00068	*HLA*-*E*, *B2M*, *HLA*-*B*	KEGG:05170
Epstein–Barr virus infection	0.00068	*HLA*-*E*, *B2M*, *HLA*-*B*	KEGG:05169
Human cytomegalovirus infection	0.00068	*HLA*-*E*, *B2M*, *HLA*-*B*	KEGG:05163
Graft-versus-host disease	0.00068	*HLA*-*E*, *HLA*-*B*	KEGG:05332
Type I diabetes mellitus	0.000696	*HLA*-*E*, *HLA*-*B*	KEGG:04940
Autoimmune thyroid disease	0.000931	*HLA*-*E*, *HLA*-*B*	KEGG:05320
Viral myocarditis	0.001095	*HLA*-*E*, *HLA*-*B*	KEGG:05416

KEGG, Kyoto Encyclopedia of Genes and Genomes.

## Data Availability

The data used to support the findings of this study are available from the corresponding author upon request.
